# Induced Salt Tolerance of Perennial Ryegrass by a Novel Bacterium Strain from the Rhizosphere of a Desert Shrub *Haloxylon ammodendron*

**DOI:** 10.3390/ijms19020469

**Published:** 2018-02-05

**Authors:** Ao-Lei He, Shu-Qi Niu, Qi Zhao, Yong-Sheng Li, Jing-Yi Gou, Hui-Juan Gao, Sheng-Zhou Suo, Jin-Lin Zhang

**Affiliations:** State Key Laboratory of Grassland Agro-Ecosystems, College of Pastoral Agriculture Science and Technology, Lanzhou University, Lanzhou 730000, China; heal15@lzu.edu.cn (A.-L.H.); niushq14@lzu.edu.cn (S.-Q.N.); qzhao@lzu.edu.cn (Q.Z.); liys@lzu.edu.cn (Y.-S.L.); goujy16@lzu.edu.cn (J.-Y.G.); gaohj15@lzu.edu.cn (H.-J.G.); suoshzh16@lzu.edu.cn (S.-Z.S.)

**Keywords:** *Haloxylon ammodendron*, rhizobacteria, perennial ryegrass, salt tolerance, complete genome sequence, *Pseudomonas* sp.

## Abstract

Drought and soil salinity reduce agricultural output worldwide. Plant-growth-promoting rhizobacteria (PGPR) can enhance plant growth and augment plant tolerance to biotic and abiotic stresses. *Haloxylon ammodendron*, a C4 perennial succulent xerohalophyte shrub with excellent drought and salt tolerance, is naturally distributed in the desert area of northwest China. In our previous work, a bacterium strain numbered as M30-35 was isolated from the rhizosphere of *H. ammodendron* in Tengger desert, Gansu province, northwest China. In current work, the effects of M30-35 inoculation on salt tolerance of perennial ryegrass were evaluated and its genome was sequenced to identify genes associated with plant growth promotion. Results showed that M30-35 significantly enhanced growth and salt tolerance of perennial ryegrass by increasing shoot fresh and dry weights, chlorophyll content, root volume, root activity, leaf catalase activity, soluble sugar and proline contents that contributed to reduced osmotic potential, tissue K^+^ content and K^+^/Na^+^ ratio, while decreasing malondialdehyde (MDA) content and relative electric conductivity (REC), especially under higher salinity. The genome of M30-35 contains 4421 protein encoding genes, 12 rRNA, 63 tRNA-encoding genes and four rRNA operons. M30-35 was initially classified as a new species in *Pseudomonas* and named as *Pseudomonas* sp. M30-35. Thirty-four genes showing homology to genes associated with PGPR traits and abiotic stress tolerance were identified in *Pseudomonas* sp. M30-35 genome, including 12 related to insoluble phosphorus solubilization, four to auxin biosynthesis, four to other process of growth promotion, seven to oxidative stress alleviation, four to salt and drought tolerance and three to cold and heat tolerance. Further study is needed to clarify the correlation between these genes from M30-35 and the salt stress alleviation of inoculated plants under salt stress. Overall, our research indicated that desert shrubs appear rich in PGPRs that can help important crops tolerate abiotic stress.

## 1. Introduction

Drought and soil salinity limit crop productivity worldwide [1,2]. Osmotic stress from high salinity exposure triggers imbalance of ions, ion toxicity-induced metabolism imbalances and a series of metabolic responses and water deficiency induced by osmotic stress in plants [3,4]. PGPR can help plants tolerate abiotic stresses [5,6]. In the last decade, researchers reported that bacteria belonging to various genera including *Rhizobium*, *Bacillus*, *Pseudomonas*, *Pantoea*, *Paenibacillus*, *Burkholderia*, *Achromobacter*, *Azospirillum*, *Microbacterium*, *Methylobacterium*, *Variovorax*, *Enterobacter*, etc. provided tolerance to host plants under different abiotic stress environments [7,8,9]. These bacteria mediated salt tolerance in plants through modulating of reactive oxygen species (ROS) scavenging enzyme expression [10], altering the selectivity of Na^+^, K^+^, and Ca^2+^ and sustaining a higher K^+^/Na^+^ ratio in plants [11]. Our previous work showed that *Bacillus subtilis* GB03 triggered upregulation of *PtHKT1;5* and *PtSOS1* but downregulation of *PtHKT2;1* in roots reduced Na^+^ transport from root to shoot as well as Na^+^ uptake in roots [12]; GB03 promoted growth in *Codonopsis pilosula* (Franch.) [13], drought tolerance in ryegrass [14] and salt tolerance in wheat [15], white clover [16] and *Codonopsis pilosula* [17]. 

Among the many known PGPR genera, *Pseudomonas* has received much research attention because it is widely distributed in various environments and is easy to culture under laboratory conditions [18]. *Pseudomonas* can survive and prosper in a wide range of environments with many strains isolated from various environments, such as soil [19], plant [20], straw [21], animal [22] and fresh and saline water [23,24]. By now, 255 species with validly published names have been described (http://www.bacterio.net). *Pseudomonas* is beneficial for plant growth promotion [25,26,27,28]. The use of these beneficial microorganisms was considered as one of the most promising methods for safe crop-management practices [29]. In order to further exploit the relevant genetic PGPR traits, complete genome sequence technology of *Pseudomonas* was widely used and many species from the genus were genetically studied [30,31,32,33,34,35,36]. *Haloxylon ammodendron*, a C4 perennial shrub, is a succulent xerohalophyte that dominantly colonizes in arid areas. It is mainly distributed in Junggar Basin, northeast of Tarim basin, Badan Jaran desert, Tengger desert and Ulanbuh desert, China, where the average annual rainfall is around 100 mm, while the average annual evaporation is over 3500 mm [37]. 

Turfgrasses are increasingly subjected to soil salinity in many areas due to the accelerated soil salinization and increasing effluent water use for irrigating turfgrass landscapes [38]. Perennial ryegrass (*Lolium perenne* L.) is one of the most popular cool-season perennial grass species with high yield and superior quality in temperate regions around the world [39,40]. Perennial ryegrass has good turf quality such as dense root system, superior tillering, and regeneration ability [41]. However, the salinity tolerance of perennial ryegrass is ranked as moderate for commercial cultivars [42]. Therefore, enhancing the perennial ryegrass to better counter salt stress is very essential for improving its growth and production. 

Therefore, exploring novel *Pseudomonas* strains is becoming more and more important.

Now that *H. ammodendron* can survive in harsh environmental conditions with strong roots, we proposed that the root system of *H. ammodendron* could provide a unique habitat for beneficial bacteria and these bacteria could help *H. ammodendron* itself and crops adapt to the extreme environment. In our previous work, we isolated over 290 bacterium strains from the rhizosphere of *H. ammodendron* in Tengger desert, Gansu province, northwestern China (unpublished data). Among these strains, we found that a strain numbered M30-35 had the ability to promote plant growth of model plant *Arabidopsis thaliana* under salt stress conditions in our preliminary experiment (unpublished data). The aims of this work were to evaluate the effects of M30-35 inoculation on salt tolerance on Perennial ryegrass and explore its genetic property as a PGPR strain by complete genome sequencing technology.

## 2. Results

### 2.1. M30-35 Promoted Ryegrass Growth under Salinity Conditions

**Figure 1:**
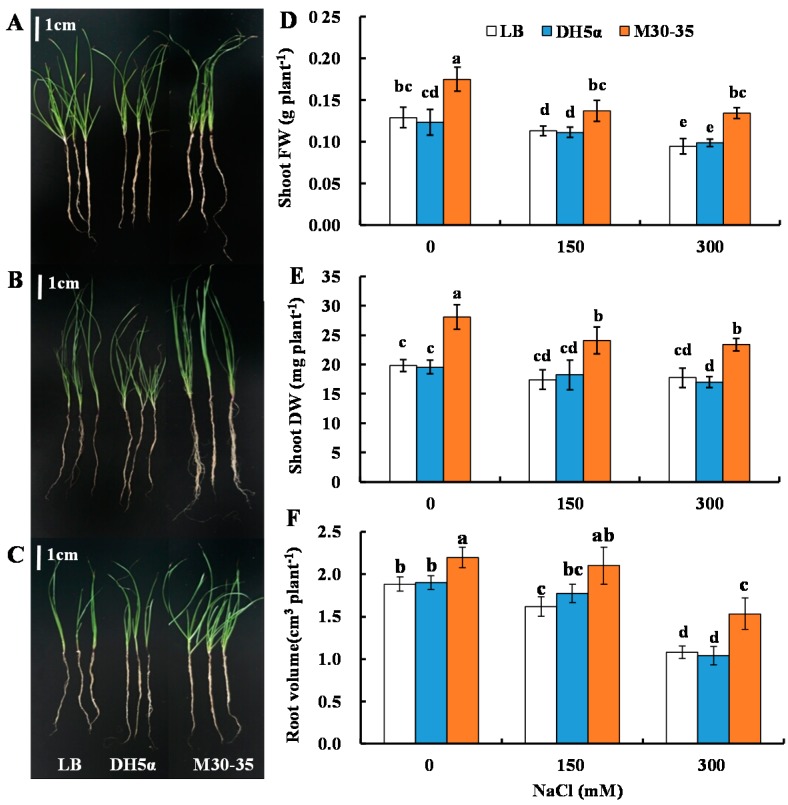
Effects of M30-35 on whole plant growth (**A**–**C**), shoot fresh weight (FW) (**D**), shoot dry weight (DW) (**E**) and root volume (**F**) of ryegrass under various salt treatments (0, 150 and 300 mM NaCl). For (**A**–**C**), from left to right: Luria broth (LB) medium, DH5α and M30-35 treatments; from top to bottom: 0, 150 and 300 mM NaCl treatments. Values are means and bars indicate standard errors (SEs) (*n* = 12). Columns with different letters indicate significant differences among treatments at *p* < 0.05 (ANOVA and Duncan’s multiple comparison test).

Statistically significant growth differences in the whole plant level were observed between M30-35 treatment and the other two treatments, *Escherichia coli* strain DH5α and Luria broth (LB) medium, after two week treatments. Plants inoculated with M30-35 had larger size than those inoculated with DH5α and LB medium (Figure 1A). Strain M30-35 enhanced shoot fresh weight, dry weight and root volume of ryegrass under both non-saline and saline stress (150 and 300 mM NaCl) (Figure 1B). Compared to LB medium (control), shoot fresh weight was significantly increased by 35%, 21% and 38%, and dry weight by 42%, 42% and 32% (*p* < 0.05) under 0, 150 and 300 mM NaCl treatments, respectively. Root volume was increased significantly by 17%, 30% and 42% (*p* < 0.05) compared to corresponding controls, respectively, under 0, 150 and 300 mM NaCl treatments. 

### 2.2. Effects of M30-35 on Chlorophyll Content under Salinity Conditions

**Figure 2:**
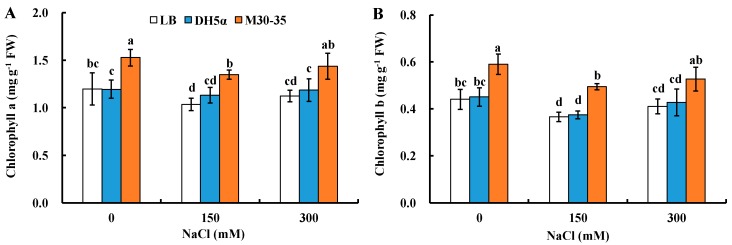
Effects of M30-35 on leaf chlorophyll a content (**A**) and leaf chlorophyll b content (**B**) of ryegrass under various salt stress (0, 150 and 300 mM NaCl). Values are means and bars indicate SEs (*n* = 12). Columns with different letters indicate significant differences among treatments at *p* < 0.05 (ANOVA and Duncan’s post hoc multiple comparison test).

After two-week treatments, in addition to promoting shoot growth, M30-35 increased both leaf chlorophyll a and chlorophyll b contents under both non-saline and salinity. Compared to corresponding controls, chlorophyll a content in plants inoculated with M30-35 was increased significantly by 28%, 30% and 28% (Figure 2A) and chlorophyll b content by 32%, 35% and 28% (*p* < 0.05) under 0, 150 and 300 mM NaCl treatments, respectively (Figure 2B).

### 2.3. Effects of M30-35 on Root Activity of Ryegrass

**Figure 3:**
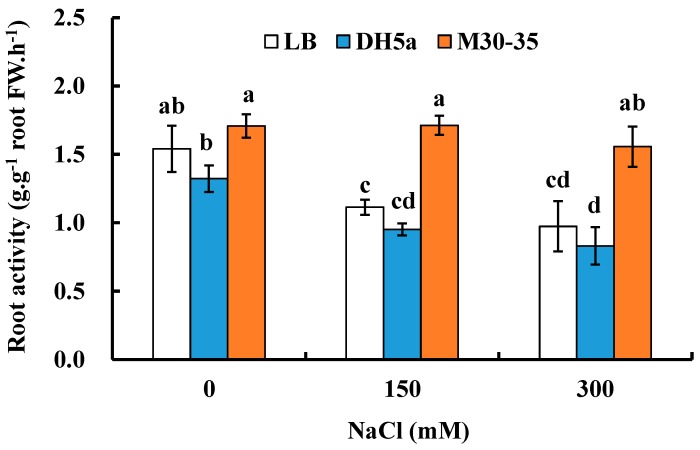
Effects of M30-35 on root activity of ryegrass under salt treatment (0, 150 and 300 mM NaCl). Values are means and bars indicate SEs (*n* = 12). Columns with different letters indicate significant differences among treatments at *p* < 0.05 (ANOVA and Duncan’s multiple comparison test).

To test if M30-35 could maintain root vigor, root activity of plants inoculated with M30-35 was significantly enhanced by 54% and 60% (*p* < 0.05) under 150 and 300 mM NaCl conditions, respectively, compared to corresponding controls (Figure 3). The above results demonstrated that M30-35 can maintain stable root vigor of ryegrass under salt stress.

### 2.4. Effects of M30-35 on Leaf Cell Membrane Integrity of Ryegrass under Salinity Conditions

**Figure 4:**
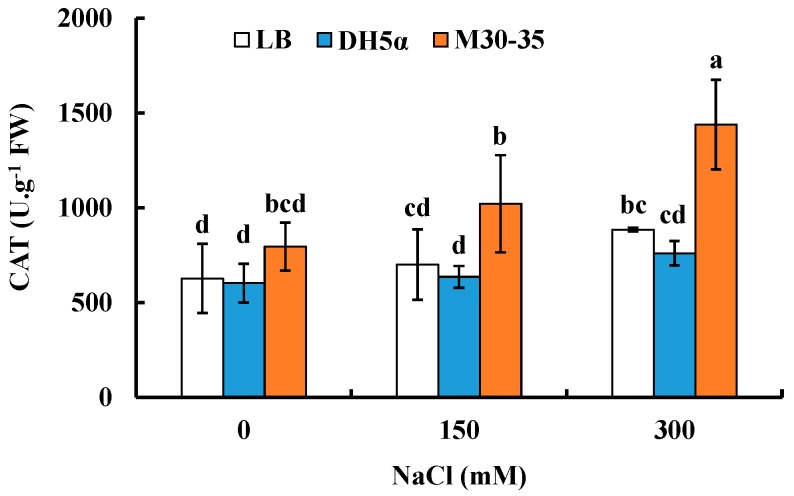
Effects of M30-35 on leaf catalase (CAT) activity of ryegrass under various salt treatment (0, 150 and 300 mM NaCl). Values are means and bars indicate SEs (*n* = 12). Columns with different letters indicate significant differences among treatments at *p* < 0.05 (ANOVA and Duncan’s multiple comparison test).

M30-35 alleviated oxidative stress of ryegrass under salt stress. After two week treatments, application of strain M30-35 significantly improved catalase (CAT) activity by 46% and 63% under salt conditions (150 and 300 mM NaCl), respectively, compared to corresponding controls (Figure 4).

**Figure 5:**
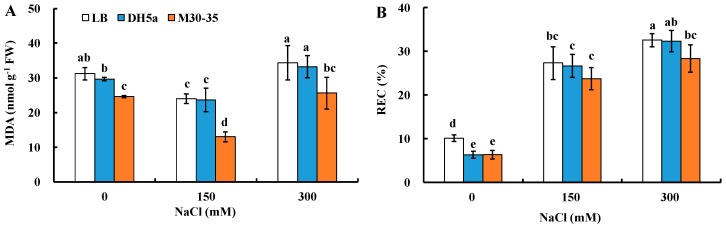
Effects of M30-35 on leaf malonyldialdehyde (MDA) content (**A**) and relative electric conductivity (REC) (**B**) of ryegrass under various salt treatments (0, 150 and 300 mM NaCl). Values are means and bars indicate SEs (*n* = 12). Columns with different letters indicate significant differences among treatments at *p* < 0.05 (ANOVA and Duncan’s multiple comparison test).

To test leaf cell membrane integrity under salt stress, malonyldialdehyde (MDA) content and relative electric conductivity (REC) were measured. After two-week treatments, MDA content in the leaves of plants inoculated with M30-35 was 21%, 84% and 34% significantly (*p* < 0.05) lower than corresponding controls under 0, 150 and 300 mM NaCl treatments, respectively (Figure 5A). Similarly, REC was 37% and 15% significantly (*p* < 0.05) lower than corresponding controls under 0 and 300 mM NaCl treatments, respectively (Figure 5B).

### 2.5. Effects of M30-35 on Leaf Osmotic Adjustment Capability of Ryegrass under Higher Salt Stress

**Figure 6:**
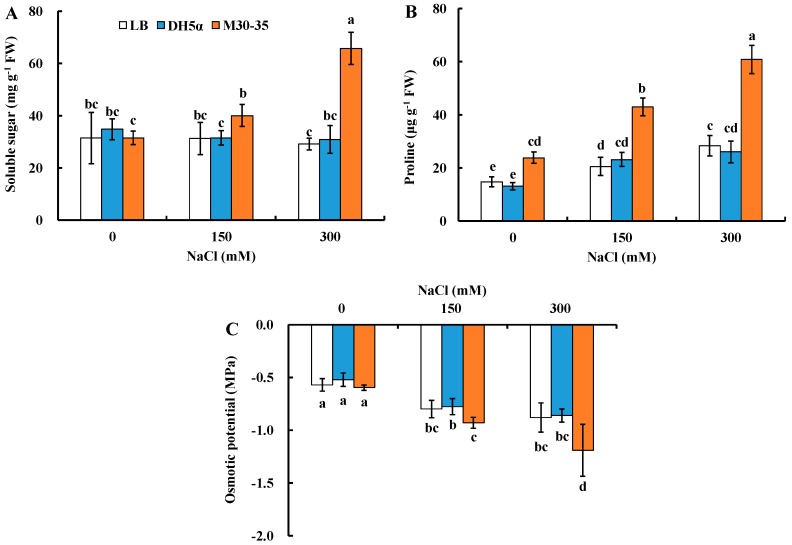
Effects of M30-35 on soluble sugar content (**A**), proline content (**B**) and osmotic potential (**C**) of ryegrass under salt treatment (0, 150 and 300 mM NaCl). Values are means and bars indicate SEs (*n* = 12). Columns with different letters indicate significant differences among treatments at *p* < 0.05 (ANOVA and Duncan’s multiple comparison test).

Leaf soluble sugar and proline are two major osmotic adjustment substances and their contents can reflect leaf osmotic adjustment capability. Under 0 and 150 mM NaCl, M30-35 had no significant effects on leaf soluble sugar content; however, it significantly enhanced leaf soluble sugar content by 1.2-fold (*p* < 0.05) compared to control under 300 mM NaCl (Figure 6A).

Leaf proline content was significantly increased by 62%, 1.1 and 1.1-fold (*p* < 0.05) by M30-35 under 0, 150 and 300 mM NaCl treatments, respectively, compared to corresponding controls (Figure 6B). 

M30-35 had no significant effects on leaf osmotic potential under 0 and 150 mM NaCl conditions; however, osmotic potential was significantly decreased by 35% (*p* < 0.05) by M30-35 under 300 mM NaCl compared to control (Figure 6C). Therefore, leaf osmotic adjustment capability of ryegrass under higher salt stress was enhanced by M30-35.

### 2.6. M30-35 Maintained K^+^/Na^+^ Ratio in Ryegrass under Salinity Conditions

To test whether M30-35 inoculation could alter ion accumulation in ryegrass under various salinity conditions, endogenous Na^+^ and K^+^ contents were measured, and K^+^/Na^+^ ratio was calculated accordingly. 

**Figure 7:**
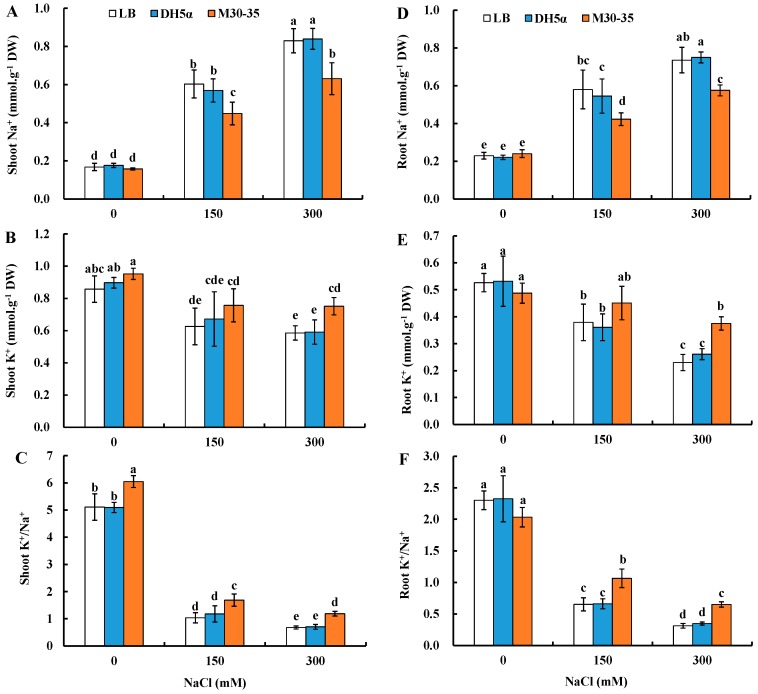
Effects of M30-35 on tissue Na^+^ (**A**,**D**) and K^+^ (**B**,**E**) contents and K^+^/Na^+^ (**C**,**F**) ratio of ryegrass under various salt conditions (0, 150 and 300 mM NaCl). Values are means and bars indicate SEs (*n* = 12). Columns with different letters indicate significant differences among treatments at *p* < 0.05 (ANOVA and Duncan’s multiple comparison test).

Compared to corresponding controls, M30-35 significantly decreased shoot Na^+^ content by 35% and 32% and root Na^+^ content by 37% and 31% (*p* < 0.05) with 150 and 300 mM NaCl treatments, respectively (Figure 7A,D). M30-35 had no significant effects on K^+^ content in both shoot and root under 0 and 150 mM NaCl; however, K^+^ content in shoot and root was significantly improved by 28% and 63% under 300 mM NaCl (*p* < 0.05) (Figure 7B,E).

Tissue K^+^/Na^+^ ratio was also significantly improved by M30-35. Compared to corresponding controls, shoot K^+^/Na^+^ ratio was significantly increased by 18%, 63% and 76% under 0, 150 and 300 mM NaCl and root K^+^/Na^+^ ratios by 63% and 1.08-fold (*p* < 0.05) under 150 and 300 mM NaCl, respectively (Figure 7C,F).

### 2.7. Complete Genome Sequence of M30-35 and Identification of Potential Genes Responsible for Plant Growth Promotion

Classification analysis and identification based on 16S rRNA gene sequence in complete genome indicated that strain M30-35 was closely related to members of the genus *Pseudomonas*. The complete genome sequence of *Pseudomonas* sp. M30-35 was deposited in GenBank under the accession number CP020892. We initially classified the strain M30-35 as a new species in *Pseudomonas* and named it as *Pseudomonas* sp. M30-35.

The genome of M30-35 was composed a circular chromosome with a size of 4,926,954 bp and an overall guanine (G) and cytosine (C) content of 54.3% with the total number of genes 4500. The chromosome contains 63 tRNA, 12 rRNA and 4 rRNA operons (Table 1).

**Figure 8:**
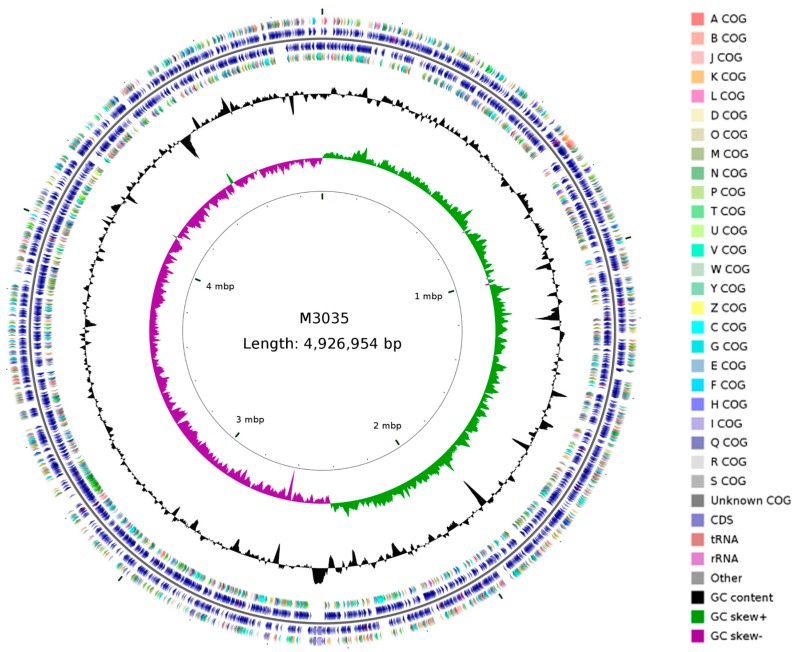
Circular representation of M30-35 genome. From the inner to outer circle: the first circle shows the scale; the second and third circles show G + C skew and G + C content, respectively; the fourth and seventh circle shows the distribution of genes related to COG categories; the fifth and sixth circle shows CDS, tRNA and rRNA on the location of the complete genome. A: RNA processing and modification; B: Chromatin structure and dynamics; J: Translation, ribosomal structure and biogenesis; K: Transcription; L: Replication, recombination and repair; D: Cell cycle control, cell division, chromosome partitioning; O: Posttranslational modification, protein turnover, chaperones; M: Cell wall, cell membrane, cell envelope biogenesis; N: Cell motility; P: Inorganic ion transport and metabolism; T: Signal transduction mechanisms; U: Intracellular trafficking, secretion, and vesicular transport; V: Defense mechanisms; W: Extracellular structures; Y: Nuclear structure; Z: Cytoskeleton; C: Energy production and conversion; G: Carbohydrate transport and metabolism; E: Amino acid transport and metabolism; F: Nucleotide transport and metabolism; H: Coenzyme transport and metabolism; I: Lipid transport and metabolism; Q: Secondary metabolites biosynthesis, transport and catabolism; R: General function prediction only; S: Unknown function.

We performed clusters of orthologous genes (COG) function classification according to Shen et al. [31] and Powell et al. [43]. Based on COG, in detail, there is one gene for RNA processing and modification, two genes for chromatin structure and dynamics, 250 genes for energy production and conversion, 31 genes for cell cycle control, cell division, chromosome partitioning, 436 genes for amino acid transport and metabolism, 78 genes for nucleotide transport and metabolism, 200 genes for carbohydrate transport and metabolism, 140 genes for coenzyme transport and metabolism, 177 genes for lipid transport and metabolism, 184 genes for translation, ribosomal structure and biogenesis, 332 genes for transcription, 136 genes for replication, recombination and repair, 211 genes for cell wall, cell membrane, cell envelope biogenesis, 94 genes for cell motility, 156 genes for posttranslational modification, protein turnover, chaperones, 221 genes for inorganic ion transport and metabolism, 120 genes for secondary metabolites biosynthesis, transport and catabolism, 528 genes for general function prediction only, 224 genes for signal transduction mechanisms, 106 genes for intracellular trafficking, secretion, and vesicular transport, 47 genes for defense mechanisms, and 337 genes for unknown function (Figure 8).

We identified 34 genes that were responsible for plant growth promotion and abiotic stress tolerance in *Pseudomonas* sp. M30-35 genome (Table 2). These included 12 genes related to insoluble phosphorus solubilization, four to auxin biosynthesis, four to other process of growth promotion, seven to oxidative stress alleviation, four to salt and drought tolerance and three to cold and heat tolerance.

## 3. Discussion

### 3.1. Pseudomonas sp. M30-35 Promoted Ryegrass Growth under Salinity Conditions

Beneficial rhizobacteria promote plant growth as well as alleviate various abiotic stresses including salinity [44] and drought [14]. PGPR species like *Azospirillum* sp. and *Pseudomonas* sp. increased the growth and biomass of canola plants by regulating the oxidative stress enzymes and essential nutrient under salinity stress [45]. The plants inoculated with *P. mendocina* had significantly greater shoot biomass than the controls, suggesting that the inoculation with selected PGPRs could be an effective tool for alleviating salinity stress in salt sensitive plants [25,46]. It was suggested that varying responses of rhizobacteria on plant root architecture indicated the specificity of plant-rhizobacterium associations [47]. Similarly, *Pseudomonas* sp. M30-35, isolated directly from the rhizosphere of *H. ammodendron*, promotes the growth of ryegrass under both control condition and salt stress through improving its root volume and root activity (Figure 1F and Figure 3).

Leaf chlorophyll content reflects plant growth [48]. Salinity inhibited photosynthesis mainly through reducing chlorophyll content, leaf area and photosystem II efficiency [16,49,50]. The mitigating effect of *P. putida* and *Bacillus subtilis* against salt stress and the increase in chlorophyll content were observed in canola and white clover seedlings, respectively [16,51]. In agreement with those observations, in our research, the significant increase in chlorophyll content by M30-35 was also observed under salt stress (0, 150, 300 mM NaCl) (Figure 2). These results implied that *Pseudomonas* sp. M30-35 plays a positive regulatory role in improving photosynthesis of ryegrass under salt treatment.

### 3.2. Pseudomonas sp. M30-35 Maintained Cell Membrane Integrity and Improved Osmotic Adjustment Capability of Ryegrass under Salinity Conditions

Abiotic stressors increase cellular levels of ROS like superoxide radical, hydroxyl radicals and hydrogen peroxide, leading to lipid peroxidation of membranes [52] and the increases in the content of the biomarker, malondialdehyde (MDA) [4,16,53] and the relative electric conductivity (REC) [14,54]. Plants have various enzymes that reduce ROS level and alleviate oxidative stress [4,53,55]. It was reported that *Bacillus subtilis* GB03 reduced MDA content in white clover under salinity [16]; root endophyte *Piriformospora indica* induced salt tolerance of barley through a strong increase in antioxidants [56]; and PGPRs enhanced abiotic stress tolerance in *Solanum tuberosum* through inducing changes in the expression of ROS-scavenging enzymes [10]. Our data showed that M30-35 inoculation significantly increased leaf CAT activity and decreased in MDA content and REC of ryegrass under higher salinity (300 mM NaCl), therefore maintaining leaf cell membrane integrity. These observations suggested that *Pseudomonas* sp. M30-35 contributed to the reduction of lipid peroxidation and the maintenance of membrane functions when plants were subjected to salinity. 

Soluble sugars (such as glucose, fructose and sucrose) have been reported to be pivotal components for osmotic adaptation when plants respond to abiotic stress [4,57], especially increasing soluble sugar content could decrease osmotic potential in cells and maintain normal physiological function of plant cells in abiotic stress conditions [58,59]. Proline is a major plant osmotic regulator with multiple functions in adjusting osmosis, stabilizing the structure of proteins and scavenging ROS under salt stress [60,61,62]. Our results showed that both soluble sugar and proline contents were significantly enhanced by M30-35 inoculation in ryegrass under salt stress, especially higher salinity (300 mM NaCl) (Figure 6); therefore, leaf osmotic potential was significantly reduced under higher salinity. These results suggested that the elevated soluble sugar and proline accumulation could contribute to the osmotic balance in perennial ryegrass against salt stress.

### 3.3. Pseudomonas sp. M30-35 Maintained Ionic Homeostasis of Ryegrass under Salinity Conditions 

Salt stress is commonly caused by high concentrations of Na^+^ in soil [2]. Salts taken up by roots were then transported into shoots, and eventually accumulated in leaves through the transpiration stream [63]. High concentration of Na^+^ in the cytoplasm disrupt the uptake of K^+^ into plant cell, which is important for the catalytic activities of many enzymes [4]. PGPRs could help plants maintain ion homeostasis and high K^+^/Na^+^ ratios in shoots by reducing Na^+^ and Cl^+^ accumulation in leaves, increasing Na^+^ exclusion via roots, and boosting the activity of high affinity K^+^ transporters [64]. Inoculation of *Bacillus subtilis* GB03 in white clover, wheat and *Codonopsis pilosula* (Franch.) Nannf. under salt stress improved their salt tolerance by reducing Na^+^ accumulation [13,15,16]. *Acinetobacter calcoaceticus* applied *Cucumis sativus* plants had reduced sodium concentration, while potassium was abundantly present under salt stress [65]. GB03-triggered upregulation of *PtHKT1;5* and *PtSOS1* and downregulation of *PtHKT2;1* in roots restricted Na^+^ transport from root into shoot as well as Na^+^ uptake into roots of the halophyte grass *Puccinellia tenuiflora* [12]. In current research, M30-35 inoculation decreased shoot and root Na^+^ concentration, but enhanced shoot and root K^+^ concentration, and thereby increased K^+^/Na^+^ ratio in both shoot and root of ryegrass under salt stress, especially under higher salinity (300 Mm NaCl) (Figure 7). The mechanism for M30-35 to regulate ion accumulation remained to be further explored.

### 3.4. Genetic Property of Pseudomonas sp. M30-35 as a PGPR Strain

Complete genome information of endophyte *Bacillus flexus* KLBMP 4941; *Pseudomonas azotoformans* S4, *P. Antarctica* PAMC 27494, *P. putida* BIRD-1 and *P. aurantiaca* strain JD37 contains genes related to plant growth promotion, biocontrol and salt tolerance [30,32,35,66]. In agreement with those observations, we also found genes that are responsible for plant growth promotion and abiotic stress tolerance the complete genome of *Pseudomonas* sp. M30-35. The specific genes and their functions are described in our research (Table 2).

Phosphorous (P) ranks second among essential plant nutrients and a major component of vital molecules [67]. Phosphorous plays an important role in metabolic processes [68]. However, the high sorption capacity of phosphate to soil particles results in a very low mobility and availability for uptake by plants [69]. In order to avoid phosphate deficiency, the environmentally unfriendly phosphate fertilizer has been widely used in agriculture worldwide, resulting in inevitable severe environmental pollution caused by P runoff [70]. Phosphate-solubilizing bacteria (PSB) exists in soil and rhizosphere and are able to release soluble phosphate from insoluble mineral phosphate [71]. The major group of phosphate solubilizing bacteria is distributed in the genus *Pseudomonas, Bacillus* and *Acinetobacter* [72]. Through various mechanisms of solubilization and mineralization, these microorganisms can convert inorganic and organic P into available form and ultimately contribute to plant growth [68]. *Acinetobacter calcoaceticus* applied plants had abundant phosphorus as compared to control in *Cucumis sativus* under salt stress [65]. In this study, 12 genes responsible for insoluble phosphorus solubilization were identified in the complete genome of *Pseudomonas* sp. M30-35, including pyruvate kinase, malate synthase, phosphoenolpyruvate carboxylase, acetate kinase, citrate synthase, Shikimate kinase, l-lactate dehydrogenase, 2-methylcitrate synthase, exopolyphosphatase, inorganic pyrophosphatase, alkaline phosphatase and NADH pyrophosphatase, respectively (Table 2). Four genes responsible for tryptophan synthesis, including tryptophan synthase α chain (*trpA*), tryptophan synthase β chain (*trpB*) and tryptophan—tRNA ligase (*trpS*) and Tryptophan 2-halogenase (*cmdE*) were also identified. These genes may play important roles in the synthesis of tryptophan and, therefore, participate in auxin indole-3-acetic acid (IAA) biosynthesis and enhance the growth of plants [73]. In addition, four other genes responsible for nitrogen fixation protein, acetolactate synthase 3 small subunit, biosynthetic arginine decarboxylase and *S*-adenosylmethionine decarboxylase proenzyme were identified, which may also contribute to growth promotion in ryegrass inoculated with M30-35.

Several studies indicated PGPRs could alleviate oxidative stress induced by salinity. Oxidative stress was mitigated by *Acinetobacter calcoaceticus* through reducing activities of catalase, peroxidase, polyphenol oxidase and total polyphenol in *Cucumis sativus* [65]. However, *Pseudomonas aeruginosa* inoculation ameliorated adverse effects of Zn stress by enhancing antioxidative enzyme activities, superoxide dismutase (SOD), peroxidase (POD) and CAT, in wheat [74]. Also in wheat, *Dietzia natronolimnaea* inoculation enhanced gene expression of various antioxidant enzymes such as ascorbate peroxidase (APX), SOD, CAT, POD, glutathione peroxidase (GPX) and glutathione reductase (GR) and higher proline content and contributed to increased tolerance to salinity stress [75]. In our research, seven genes responsible for oxidative stress alleviation were identified in the complete genome of *Pseudomonas* sp. M30-35, including catalase, superoxide dismutase, glutathione *S*-transferase, glutathione peroxidase, glutathione reductase, *S*-(hydroxymethyl) glutathione dehydrogenase and glutathione synthetase. Further study is needed to clarify the correlation between these genes and the oxidative stress alleviation in ryegrass inoculated with *Pseudomonas* sp. M30-35 under salt stress. 

Seven genes responsible for abiotic stress in the complete genome of *Pseudomonas* sp. M30-35 were also identified, including Na^+^/H^+^ antiporter (*nhaC*), glycine betaine transporter (*opuD*), trehalose/maltose-binding protein, 1-aminocyclopropane-1-carboxylate (ACC) deaminase, cold shock protein (*capB*), cold shock protein (*cspA*) and heat shock protein (*hs1R*) (Table 2). Trehalose/maltose-binding protein can alleviate different stress conditions (dehydration, heat and cold) of plant growth environment [76]. ACC deaminase could lower the concentration of plant ethylene and decrease the deleterious effect of abiotic stress [77].

## 4. Materials and Methods

### 4.1. Bacterial Suspension Culture

*Pseudomonas* sp. strain M30-35 and *Escherichia coli* strain DH5α as a positive control bacterium strain were grown in liquid Luria broth (LB) medium without light for 24 h at 28 °C with 250 rpm rotation to yield 10^9^ colony forming units (CFU) mL^−1^, as determined by optical density and serial dilutions. Strain M30-35 was in our laboratory at Lanzhou University, Lanzhou, China, and *E. coli* strain DH5α was purchased from Takara Biotechnology (Dalian) Co., Ltd., Dalian, China.

### 4.2. Plant Growth and Treatments

Perennial ryegrass (*Lolium perenne* L. cv. Esquire) seeds (from Beijing Top Green Seed Co., Ltd., Beijing, China) were surface sterilized with 2% NaClO for 1 min followed by 70% ethanol for 10 min, and rinsed with sterile water five times. Seeds were then sown in pre-sterilized plastic pots (diameter 20 cm, depth 15 cm, 1.0 g seeds/pot) containing 1800× *g* of heat-sterilized (95 °C, 72 h) vermiculite and sand mix (volume ratio 1:1) and watered with half strength Hoagland’s nutrient solution (including 2 mM KNO_3_, 0.5 mM NH_4_H_2_PO_4_, 0.1 mM Ca(NO_3_)_2_·4H_2_O, 0.25 mM MgSO_4_·7H_2_O, 0.5 mM Fe citrate, 92 μM H_3_BO_3_, 18 μM MnCl_2_·4H_2_O, 1.6 μM ZnSO_4_·7H_2_O, 0.6 μM CuSO_4_·5H_2_O, and 0.7 μM (NH_4_)_6_Mo_7_O_24_·4H_2_O) once per week. After seed germination, each pot was inoculated with 10 mL bacterial suspension culture of M30-35 or DH5α, or 10 mL liquid LB medium as control. Plants were grown in a glasshouse at the temperature regulated to around 28 °C during the day and 23 °C at night. Relative humidity averaged 65% and 75% for day and night periods, respectively. The photoperiod was 16 h/8 h (light/dark).

Twenty days after germination, seedlings were watered with 0, 150 or 300 mM NaCl as salt treatments. Two weeks after salt treatments, plants were harvested for biomass and physiological index measurements (12 replications for each treatment).

### 4.3. Plant Biomass and Physiological Measurements

Two weeks after salt treatments, plants were removed from the pots and roots were water rinsed to remove attached soil. Root and shoot were separated and blotted gently. Shoot fresh weights and root volume were determined immediately and samples were oven dried at 80 °C for 3 days to obtain dry weights.

Leaf chlorophyll content was measured using acetone and alcohol method with slight modification [16]. Briefly, fresh leaf sample was ground thoroughly with 80% acetone (5 mL) and 95% alcohol (5 mL) as a solvent in the dark and centrifuged at 9000× *g* for 10 min at 4 °C. Absorbance reading (UV-2102C Spectrophotometer, Unico Instrument Co., Ltd., Shanghai, China) at 645 and 663 nm for collected supernatant was used to estimate chlorophyll a and chlorophyll b contents, respectively. Leaf chlorophylls content was calculated according to the formula: Chlorophyll a content = (12.72A_663_ − 2.59A_645_) × 10 ÷ 1000 ÷ W; Chlorophyll b content = (22.88 A_645_ − 4.67A_663_) × 10 ÷ 1000 ÷ W. W: fresh weight of leaves; A_663_ represented the absorbance value at 663 nm; A_645_ represented the absorbance value at 645 nm.

Root activity was measured according to the triphenyltetrazolium chloride (TTC) reduction method [78]. Fresh root tissue (0.1 g) was soaked in 10 mL reaction solution (5 mL 0.4% TTC + 5 mL phosphate buffer, pH = 7.0) at 37 °C for 3 h, and then 2 mL of 1 mol L^−1^ sulfuric acid was added to stop the reaction. Roots were taken out from the reaction solution, blotted and grounded with 3 mL ethyl acetate. Then, the supernatant was collected by ethyl acetate to the final volume of 10. Absorbance reading (UV-2102C Spectrophotometer, Unico Instrument Co., Ltd., Shanghai, China) at 485 nm of collected supernatant was used to estimate root activity. The standard curve for reduction value of TTC (*y*): *y* = 0.0015*x* + 0.0053, *R*^2^ = 0.9914 and *y* represented absorbance reading. Root activity = reduction value of TTC (*x*) ÷ W × time. W represented fresh weight of root and time 3 h.

Catalase (CAT) activity was measured according to [55]. Briefly, 0.5 g fresh leaf sample was grounded with 3 mL cold (4 °C) phosphate buffer (pH = 7.0) and enzyme was extracted in a total volume of 10 mL. CAT activity was estimated by the decrease in H_2_O_2_ according to the absorbance at 240 nm. CAT = 678 × ΔA ÷ W, ∆A = A_1_ − A_2_, A_1_ represented the initial absorbance value at 240 nm, A_2_ absorbance value after one minute and W fresh weight of leaves.

The level of lipid peroxidation in leaves was assessed by measuring the content of malondialdehyde (MDA) using the thiobarbituric acid reaction method [12,14]. Absorbance was determined at 532 (A_532_) and 600 (A_600_) nm using a UV spectrophotometer (UV-2102C, Unico Instrument Co., Ltd., Shanghai, China) to estimate MDA content. MDA = 25.8 × ΔA ÷ W. ∆A = A_532_ − A_600_ and W represented fresh weight of leaves.

To estimate leaf cell membrane damage, leaf relative electric conductivity (REC) was measured using an electric conductivity meter EC215 (Hanna Instruments Romania Srl, Nusfalau, Romania) [12]. REC (%) was calculated the following equation, REC (%) = (S1/S2) × 100, where S1 and S2 refer to electric conductivity of live leaves and boiled leaves, respectively.

The soluble sugar content was measured according to the method of anthrone colorimetry [79]. In addition, 1 g fresh leaf sample was grounded thoroughly with water and centrifuged at 8000× *g* for 10 min at 25 °C to extract soluble sugar in 10 mL. Then, 1 mL supernatant, 0.5 mL anthrone and 5 mL H_2_SO_4_ were mixed slowly and kept for 10 min. After cooling down to room temperature, absorbance was determined at 620 nm using a UV spectrophotometer (UV-2102C, Unico Instrument Co., Ltd., Shanghai, China) to estimate soluble sugar content. Soluble sugar content = 1.17 × (ΔA + 0.07) ÷ W. ΔA = A_1_ − A_0_, A_1_ represented the absorbance value of sample at 620 nm, A_0_ represented the absorbance value of blank solution at 620 nm and W fresh weight of leaves.

Leaf proline content was determined by the sulfosalicylic acid method described by [80]. Proline was extracted from 0.2 g of fresh leaf tissue into 10 mL of 3% sulfosalicylic acid and filtered through filter paper and absorbance was determined at 520 nm (A_520_) using a spectrophotometer (UV-2102C Spectrophotometer, Unico Instrument Co., Ltd., Shanghai, China). Proline content = 19.2 × (A_520_ + 0.0021) ÷ W and W represented fresh weight of leaves.

CAT, MDA, the soluble sugar and proline content were measured using reagent kit (Suzhou Comin Biotechnology Co., Ltd., Suzhou, China), and the specific steps of all methods were operated according to the instructions provided by the company.

Leaf osmotic potential (ψ_s_) was measured according to Ma et al. [48]. Fresh leaf samples were frozen in liquid nitrogen. Cell sap was collected by thawing slowly and then ψ_s_ was determined using a cryoscopic osmometer (Osmomat-030, Gonotec GmbH, Berlin, Germany) at 25 °C. The readings (mol L^−1^) were used to calculate the solute potential (ψ_s_) in MPa with the formula ψ_s_ = −The readings × *R* × *T*, here *R* = 0.008314 MPa L mol^−1^ K^−1^ and *T* = 298.8 K.

Na^+^ and K^+^ contents were measured according to the method described by Han et al. [16]. For ion content analysis, plants were harvested two weeks after bacterial inoculation. Roots were washed twice for 8 min in ice-cold 20 mM CaCl_2_ to exchange cell-wall-bound K^+^ and Na^+^, and the shoots were rinsed in deionised water to remove surface salts. Roots and shoots were separated and samples were oven dried at 80 °C for 3 days. Ions were extracted from dried tissues with 10 mL 100 mM acetic acid at 90 °C for 2 h. Ion analysis was conducted by an atomic absorption spectrophotometry r (2655-00, Cole-Parmer Instrument Co., Vernon Hills, IL, USA).

### 4.4. Data Analysis

Results of the growth and physiological parameters were presented as means with standard errors (*n* = 12). All the data were subjected to one-way analysis of variance (ANOVA) and Duncan’s multiple comparison tests were used to detect significant differences among means at a significance level of *p* < 0.05 by SPSS 19.0 (SPSS Inc., Chicago, IL, USA).

### 4.5. Complete Genome Sequencing and Analysis of M30-35

Complete genome of strain M30-35 was sequenced by Illumin Miseq, Pacific Biosciences (PacBio) RS II and Single Molecule Real Time (SMRT) sequencing technology. The complete genome sequencing work was in cooperation with Shanghai Personal Biotechnology Co., Ltd., Shanghai, China.

The genome annotation was performed using NCBI Prokaryotic Genome Annotation Pipeline (PGAP) version 4.10 (National Center for Biotechnology Information, National Library of Medicine, Bethesda, MD, USA). The analysis of complete genome was conducted at https://www.ncbi.nlm.nih.gov/genome/microbes/ and sequence blasting at https://blast.ncbi.nlm.nih.gov/Blast.cgi. The open reading frames (ORFs) were predicted using Glimmer version 3.0 (currently supported by National Library of Medicine, Bethesda, MD, USA) [31,32], tRNA and rRNA genes were identified by tRNAscan-SE version 1.3.1 (The Lowe Lab, Biomolecular Engineering, University of California Santa Cruz, Santa Cruz, CA, USA) [33,35] and rRNAmmer version 1.2 (Department of Bio. and Health Informatics, Technical University of Denmark Bioinformatics, Lyngby, Denmark) [81], respectively. We also performed functional analysis and classification of the clusters of orthologous genes (COG) [43].

## 5. Conclusions

*Pseudomonas* sp. M30-35 live naturally in the rhizosphere desert shrub and xerohalophyte *Haloxylon ammodendron*. Our study firstly demonstrated that M30-35 had the feature as a PGPR strain. The soil inoculation of M30-35 significantly enhanced growth and salt tolerance of perennial ryegrass through increasing chlorophyll content, root activity, catalase activity, soluble sugar content, proline content, tissue K^+^ content and K^+^/Na^+^ ratio, while decreasing malondialdehyde content and relative electric conductivity and osmotic potential under salt condition, especially higher salinity. M30-35 was initially classified as a new species in *Pseudomonas* and named it as *Pseudomonas* sp. M30-35. Thirty-four genes, which were responsible for plant growth promotion and abiotic stress tolerance in *Pseudomonas* sp. M30-35 genome, were identified through complete genome sequencing technology. Further study is needed to clarify the correlation between these genes from M30-35 and the salt stress alleviation in inoculated plants under salt stress. Genome sequencing and physiological analysis in our study indicated that *Pseudomonas* sp. M30-35 is a novel PGPR strain. Moreover, this work will be useful to explore novel PGPR strains from rhizosphere of plants under extreme environments and the potential interaction mechanism between PGPRs strains and crop plants.

## Figures and Tables

**Table 1:** Genome features of *Pseudomonas* sp. M30-35.

Features	Values
Genome size (bp)	4,926,954
Guanine (G) + cytosine (C) content (%)	54.3
Total number of genes	4500
Number of proteins	4364
Protein encoding genes	4421
rRNAs (5S, 16S, 23S)	12
tRNAs	63
rRNA operons	4
ncRNA	4
Pseudogenes	57

**Table 2:** Potential genes responsible for plant growth promotion and stress tolerance in *Pseudomonas* sp. M30-35 Genome.

Categories	Gene ID	Gene Annotation
		**Insoluble phosphorus solubilization**
**Plant growth promotion**	ORF03861	Pyruvate kinase
	ORF00725	Malate synthase
	ORF01978	Phosphoenolpyruvate carboxylase
	ORF04759	Acetate kinase
	ORF03358	Citrate synthase
	ORF05557	Shikimate kinase
	ORF00482	llactate dehydrogenase
	ORF02892	2-methylcitrate synthase
	ORF05912	Exopolyphosphatase
	ORF01099	Inorganic pyrophosphatase
	ORF05696	Alkaline phosphatase
	ORF02576	Nicotinamide adenine dinucleotide (NADH) pyrophosphatase
		**Auxin biosynthesis**
	ORF00180	Tryptophan synthase α chain (*trpA*)
	ORF00181	Tryptophan synthase β chain (*trpB*)
	ORF02417	Tryptophan-tRNA ligase (*trpS*)
	ORF05537	Tryptophan 2-halogenase (*cmdE*)
		**Others related to plant growth promotion**
	ORF03057	Nitrogen fixation protein (*fixG* and *anfA*)
	ORF01526	Acetolactate synthase 3 small subunit (*ilvH*)
	ORF05343	Biosynthetic arginine decarboxylase (*speA*)
	ORF00855	*S*-adenosylmethionine decarboxylase proenzyme (*speD*)
**Stress tolerance**		**Oxidative stress alleviation**
	ORF00943	Catalase
	ORF04857	Superoxide dismutase
	ORF03162	Glutathione *S*-transferase
	ORF02859	Glutathione peroxidase
	ORF03063	Glutathione reductase
	ORF04403	*S*-(hydroxymethyl) glutathione dehydrogenase
	ORF00537	Glutathione synthetase
		**Salt and drought tolerance**
	ORF04529	Na^+^/H^+^ antiporter (*nhaC*)
	ORF05136	Glycine betaine transporter (*opuD*)
	ORF00154	Trehalose/maltose-binding protein
	ORF04015	1-aminocyclopropane-1-carboxylate (ACC) deaminase
		**Cold and heat shock protein**
	ORF01759	Cold shock protein (*capB*)
	ORF02091	Cold shock protein (*cspA*)
	ORF00761	Heat shock protein (*hs1R*)

## Abbreviations

ACC	1-aminocyclopropane-1-carboxylate
APX	Ascorbate peroxidase
CAT	Catalase
CFU	Colony forming unit
COG	Clusters of orthologous gene
G + C	Guanine and Cytosine
GDH	Glucose dehydrogenase
GPX	Glutathione peroxidase
GR	Glutathione reductase
LB	Luria broth
MDA	Malondialdehyde
NADH	Nicotinamide adenine dinucleotide
PGAP	Prokaryotic genome annotation pipeline
PGPR	Plant growth promoting rhizobacterium
POD	Peroxidase
PSB	Phosphate solubilizing bacterium
REC	Relative electric conductivity
ROS	Reactive oxygen species
SEs	Standard errors
SMRT	Single molecule real time
SOD	Superoxide dismutase
TBA	Thiobarbituric acid
TTC	Triphenyltetrazolium chloride
